# An intron polymorphism of the fibronectin gene is associated with end-stage knee osteoarthritis in a Han Chinese population: two independent case**-**control studies

**DOI:** 10.1186/1471-2474-15-173

**Published:** 2014-05-23

**Authors:** Hsin-Yi Yang, Sui-Lung Su, Yi-Jen Peng, Chih-Chien Wang, Herng-Sheng Lee, Donald M Salter, Chian-Her Lee

**Affiliations:** 1 School of Public Health, National Defense Medical Center, Taipei, Taiwan; 2 Department of Pathology, Tri-Service General Hospital, National Defense Medical Center, Taipei, Taiwan; 3 Department of Orthopedics, Tri-Service General Hospital and National Defense Medical Center, Taipei, Taiwan; 4 Graduate Institute of Medical Sciences, National Defense Medical Center, Taipei, Taiwan; 5 Centre for Molecular Medicine, MRC IGMM, University of Edinburgh, Edinburgh, UK; 6 Department of Orthopedics, School of Medicine, College of Medicine, Taipei Medical University and Hospital, No.250, Wuxing St., Xinyi Dist, Taipei, Taiwan

**Keywords:** Knee osteoarthritis, Fibronectin gene, Integrin αV gene, Single nucleotide polymorphism

## Abstract

**Background:**

Knee osteoarthritis (OA) is a complex disease involving both biomechanical and metabolic factors that alter the tissue homeostasis of articular cartilage and subchondral bone. The catabolic activities of extracellular matrix degradation products, especially fibronectin (FN), have been implicated in mediating cartilage degradation. Chondrocytes express several members of the integrin family which can serve as receptors for FN including integrins α5β1, αvβ3, and αvβ5. The purpose of this study was to determine whether polymorphisms in the FN (*FN-1*) and integrin genes are markers of susceptibility to, or severity of, knee OA in a Han Chinese population.

**Methods:**

Two independent case–control studies were conducted on 928 patients with knee OA and 693 healthy controls. Ten single nucleotide polymorphisms (SNPs) of *FN-1* and the integrin αV gene (*ITGAV*) were detected using the ABI 7500 real-time PCR system.

**Results:**

The AT heterozygote in *FN-1* (rs940739A/T) was found to be significantly associated with knee OA (adjusted OR = 1.44; 95% CI = 1.16–1.80) in both stages of the study. *FN-1* rs6725958C/A and *ITGAV* rs10174098A/G SNPs were only associated with knee OA when both study groups were combined. Stratifying the participants by Kellgren-Lawrence (KL) score identified significant differences in the *FN-1* rs6725958C/A and rs940739 A/T genotypes between patients with grade 4 OA and controls. Haplotype analyses revealed that TGA and TAA were associated with a higher risk of OA, and that TAG conferred a lower risk of knee OA in the combined population.

**Conclusions:**

Our study suggests that the *FN-1* rs940739A/T polymorphism may be an important risk factor of genetic susceptibility to knee OA in the Han Chinese population.

## Background

Osteoarthritis (OA) is a degenerative joint disease that progressively causes loss of joint function and is a leading cause of disability and impaired quality of life among the elderly in developed countries. It is characterized by degeneration and the progressive loss of articular cartilage with pathological changes in bone, synovium, and other soft tissues of affected joints [1]. A number of factors, such as genetic predisposition, aging, obesity, inflammation, and excessive mechanical loading, are recognized to contribute to OA onset and progression [2]. As such, OA is a complex disease involving both biomechanical and metabolic factors that alter the tissue homeostasis of articular cartilage and subchondral bone [3].

The catabolic activities of matrix degradation products, including fibronectin fragments (FN-fs), have been implicated in mediating cartilage degradation [4,5]. At least some of these fragments appear to act via an integrin-dependent mechanism [6]. Chondrocytes express several members of the integrin family that can serve as FN receptors, including α5β1, αvβ3, and αvβ5 [7,8].

FN is encoded by *FN-1*, which is located on chromosome 2q34-36, is over 75 kb in length and is composed of 50 exons. FN is a multifunctional glycoprotein that is involved in a wide range of biological processes such as cell migration, wound healing, angiogenesis, and differentiation. FN exists in two major forms. The soluble form is found in the plasma, while the insoluble form is present in tissues, including at low levels in the extracellular matrix (ECM) of normal cartilage [9]. Alternative splicing of two type III exons, known as Extra Domains A and B, and a variable region result in the production of up to 20 different FN variants [10]. Growing evidence suggests that these isoforms affect the function of FN and FN-fs, including cartilage breakdown in OA [11].

Integrins are cell surface receptors composed of α and β chains. The integrin family consists of at least 24 different heterodimers that are involved in cell–cell and cell–ECM adhesion [12]. Integrin αV (ITGAV) is one of the more promiscuous alpha integrins associating with five different beta subunits including β1, β3, β5, β6, and β8 [13]. Expression of ITGAV appears to be upregulated in osteoarthritic chondrocytes [14].

Single nucleotide polymorphisms (SNPs) represent variations in the genome between individuals. Several studies have demonstrated that a number of these polymorphisms are associated with OA and may contribute to the genetic risk of developing the disease [15-17]. Because *FN-1* and *ITGAV* polymorphisms may influence gene expression or modify the biological activities of the functional protein, we set out to test the hypothesis that such polymorphisms influence susceptibility and the severity of knee OA.

## Methods

A total of 928 patients with knee OA and 693 healthy individuals were recruited for two independent studies carried out between July 2008 and December 2009 (the first study conducted in Miaoli, which is located in a largely rural area of China) and between March 2010 and June 2011 (the second study conducted in the centrally located Chinese city of Taipei). The first study recruited and genotyped 403 knee OA patients (65.4% female) and 314 controls (53.8% female) to identify knee OA-associated SNPs and haplotypes. The second study recruited and genotyped 525 knee OA patients (65.6% female) and 379 healthy controls (58.6% female).

Disease severity in the knee OA populations, who were attending an orthopedic hospital, was assessed by Kellgren-Lawrence (KL) grading. All patients had a KL score ≥ 2. Other etiologies of knee joint disease such as inflammatory arthritis, post-traumatic or post-septic arthritis, and skeletal or developmental dysplasia were excluded from the study. Healthy control subjects had no signs or symptoms of joint disease (pain, swelling, tenderness, or restriction of movement) and standard x-rays of the knee joints confirmed an absence of OA. This study was reviewed and approved by the institutional ethical committee of Taipei Medical University Hospital and Tri-Service General Hospital (CRC-01-10-03 and TSGH-100-05-023). All clinical and biological samples were collected and DNA was genotyped following approval by this committee. Written informed consent was obtained from all participants after the study had been fully explained to them.

### Selection and genotyping of polymorphisms

We selected *FN-1* and *ITGAV* as candidate genes based on the published literature [18,19]. SNP genotype information was downloaded from the HapMap database (http://hapmap.ncbi.nlm.nih.gov/) and The National Center for Biotechnology Information dbSNP database (http://www.ncbi.nlm.nih.gov/snp) to select the most representative SNPs by capturing the majority of genetic variation. Using the tagger program implemented in Haploview 4.0, tag SNPs across *FN-1* and *ITGAV* were selected on the basis of linkage disequilibrium patterns observed in the Han Chinese samples genotyped as part of the International HapMap Project. Only SNPs with a minor allele frequency greater than 5% in HapMap were considered. Six SNPs (rs10202709, rs6725958, rs940739, rs2304573, rs11651, and rs3796123) in *FN-1* and four SNPs (rs3911238, rs10174098, rs3738929, and rs1448427) in *ITGAV* were included to capture as much variation as possible. These 10 tag SNPs captured all alleles with an r^2^ of at least 0.8.

Genomic DNA was extracted from the peripheral blood of patients and controls using the QIAamp DNA Blood Mini Kit (QIAGEN Inc., Hilden, Germany) according to the manufacturer’s instructions. Genotyping was performed using the TaqMan® SNP Genotyping Assay (Applied Biosystems, Foster City, CA, USA) with the following cycling conditions: 95°C for 10 min, followed by 40 cycles of 95°C for 15 s and 60°C for 1 min, with a 1 min extension at 25°C following the last cycle. Genotyping was performed by laboratory personnel blinded to case status and 10% of the samples were randomly selected for repeated testing to validate genotyping procedures. Two authors independently reviewed the genotyping results, data entry, and statistical analyses.

### Statistical analysis

Statistical analysis was performed using SPSS for Windows version 18.0 software (SPSS, Chicago, IL, USA), and results were considered statistically significant where the two-tailed *p*-value was less than 0.05. SNP deviations from the Hardy-Weinberg equilibrium (HWE) in control samples were assessed using the standard chi-squared (χ^2^) test. The allele and genotype frequencies of OA patients and control subjects were compared using χ^2^ statistics or Fisher’s exact test as appropriate. The demographics were evaluated by the Student’s *t*-test or Mann–Whitney U test for continuous variables and expressed as means ± standard deviation (SD). Logistic regression was used to estimate crude and adjusted age, gender, and body mass index (BMI) odd ratios (ORs) and 95% confidence intervals (CIs) as a measure of association with the risk of OA.

Linkage disequilibrium and haplotype analyses were performed using Haploview software (http://www.broad.mit.edu/mpg/haploview/) [20]. Because the frequency distributions of demographic characteristics and genotypic and allelic distributions of the 10 SNPs were similar between the two study cohorts, all data were pooled to analyze the association between SNPs and knee OA. In the absence of interstudy heterogeneity within samples, we also constructed a Mantel-Haenszel meta-analysis of sample data to assess the overall evidence of association. The Mantel-Haenszel χ^2^ test and estimate of the OR were computed with or without the inclusion of covariates using R, version 3.0.2, with the “metafor” and “meta” packages.

The assumption of heterogeneity for each analysis was tested using the DerSimonian-Laird method. The level of significance was determined by Bonferroni’s method for correcting multiple testing errors. For the 10 selected SNPs, a *p*-value < 0.005 (0.05 divided by 10) was considered statistically significant. In this study, power estimation was performed using CaTS (http://www.sph.umich.edu/csg/abecasis/CaTS/) and is summarized in Additional file 1: Table S1.

## Results

### Characteristics of study subjects

The frequency distributions of demographic characteristics between the 928 knee OA cases and 693 healthy controls in the two independent studies are shown in Table 1. The average age of knee OA cases was significantly higher than the control population. The proportion of females in the knee OA cases (65.5%) was significantly higher than in the healthy controls (56.4%). BMI was also slightly higher in the knee OA cases.

**Table 1:** Characteristics of OA cases and healthy controls

**Variables**	**Study 1**		**Study 2**		**Study 1 + Study 2**		
	**Case (%)**	**Control (%)**	**Case (%)**	**Control (%)**	**Case (%)**	**Control (%)**	***p***
Number	403	314	525	379	928	693	
Age (mean ± SD)	72.54 ± 8.13	69.34 ± 9.80	72.18 ± 8.12	69.87 ± 9.96	72.34 ± 8.13	69.63 ± 9.88	< 0.001
Gender (% Female)	65.4%	53.8%	65.6%	58.6%	65.5%	56.4%	< 0.001
BMI (mean ± SD)	25.28 ± 3.13	24.95 ± 9.88	25.08 ± 3.16	24.26 ± 3.79	25.17 ± 3.15	24.57 ± 7.22	0.04
K-L grading (%)							
Grade 0	0	162 (51.60)	0	189 (49.87)	0	351 (50.65)	
Grade 1	0	152 (48.40)	0	190 (50.13)	0	342 (49.35)	
Grade 2	146 (36.23)	0	205 (39.05)	0	351 (37.82)	0	
Grade 3	73 (18.11)	0	74 (14.10)	0	147 (15.84)	0	
Grade 4	184 (45.66)	0	246 (46.85)	0	430 (46.34)	0	

### Association analyses of FN-1 polymorphisms with susceptibility to OA

Table 2 shows the genotype distributions and allele frequencies of *FN-1* polymorphisms in knee OA and control subjects from both studies. The genotype frequencies of all six *FN-1* SNPs were in HWE (*p* > 0.05). In study 1, the genotype distribution of *FN-1* rs940739A/T was significantly different between knee OA patients and healthy controls (*p* < 0.05). When the *FN-1* rs940739AA genotype was used as the reference group, the rs940739AT genotype appeared to be associated with a higher risk for knee OA (adjusted OR = 1.44, 95% CI = 1.03–2.01). To determine whether the results could be replicated, we genotyped the six SNPs in the second study population. In this study group, rs940739AT also differed significantly in genotype distribution between the knee OA cases and healthy controls (adjusted OR = 1.48, 95% CI = 1.10–1.99).

**Table 2:** Genotype distributions of FN-1 polymorphisms and their association with OA risk

**SNP**		**Study 1**			**Study 2**		
		**Case**	**Control**	**Adjusted OR (95% CI) **^**#**^	**Case**	**Control**	**Adjusted OR (95% CI) **^**#**^
rs10202709	GG	376	300	1	488	351	1
	GA	26	13	1.64 (0.81-3.33)	35	27	0.88 (0.52-1.52)
	AA	1	1	1.00 (0.06-16.72)	2	1	1.24 (0.11-14.25)
	G	0.96	0.98	1	0.96	0.96	1
	A	0.04	0.02	1.54 (0.80-2.97)	0.04	0.04	0.94 (0.55-1.52)
rs6725958	CC	113	102	1	120	111	1
	CA	200	158	1.08 (0.76-1.54)	289	197	1.25 (0.90-1.72)
	AA	90	54	1.44 (0.92-2.25)	116	71	1.37 (0.92-2.05)
	C	0.53	0.58	1	0.50	0.55	1
	A	0.47	0.42	1.18 (0.95-1.47)	0.50	0.45	1.16 (0.96-1.41)
rs940739	AA	240	208	1	310	260	1
	AT	146	94	1.44 (1.03-2.01)^*^	191	104	1.48 (1.10-1.99)^*^
	TT	17	12	1.07 (0.49-2.36)	24	15	1.15 (0.58-2.28)
	A	0.78	0.81	1	0.77	0.82	1
	T	0.22	0.19	1.26 (0.96-1.65)	0.23	0.18	1.30 (1.02-1.65)^*^
rs2304573	GG	227	173	1	302	212	1
	GA	146	119	0.95 (0.68-1.31)	189	137	0.99 (0.74-1.33)
	AA	30	22	1.15 (0.63-2.10)	34	30	0.80 (0.47-1.36)
	G	0.74	0.74	1	0.75	0.74	1
	A	0.26	0.26	1.02 (0.79-1.30)	0.25	0.26	0.93 (0.75-1.17)
rs11651	AA	190	138	1	251	167	1
	AG	170	130	0.93 (0.67-1.29)	213	164	0.89 (0.66-1.19)
	GG	43	46	0.71 (0.44-1.16)	61	48	0.85 (0.55-1.31)
	A	0.68	0.65	1	0.68	0.66	1
	G	0.32	0.35	0.86 (0.68-1.08)	0.32	0.34	0.91 (0.74-1.11)
rs3796123	TT	338	259	1	430	318	1
	TA	61	52	0.83 (0.54-1.27)	87	60	1.07 (0.74-1.54)
	AA	4	3	1.02 (0.22-4.68)	8	1	6.17 (0.73-52.51)
	T	0.91	0.91	1	0.90	0.92	1
	A	0.09	0.09	0.87 (0.59-1.27)	0.10	0.08	1.21 (0.86-1.70)

^*^p < 0.05; ^#^Data have been adjusted by gender, age and BMI.

The other *FN-1* SNPs (rs10202709G/A, rs6725958C/A, rs2304573G/A, rs11651A/G, and rs3796123T/A) demonstrated no significant genotypic or allelic association between OA cases and healthy controls in either of the two study groups (Table 2).

### Association analyses for FN-1 polymorphisms in the combined studies

When data from the two independent studies were combined, the association of the rs940739A/T polymorphism with knee OA was maintained (adjusted OR = 1.44, 95% CI = 1.16–1.80, *p* = 0.001). Additionally, the minor allele of *FN-1* rs6725958C/A appeared to be associated with a significantly higher risk of knee OA (adjusted OR = 1.17, 95% CI = 1.01–1.80, *p* = 0.033) (Table 3). After correcting for multiple comparisons, the *FN-1* rs940739TA genotype had a higher risk for OA, but the minor allele of *FN-1* rs6725958C/A was not significant.

**Table 3:** **Association analyses for *FN-1 *SNPs with OA in the combined analyses of both study groups**

**SNP**		**Case**	**Control**	**Simply pooled OR (95% CI)**^**#**^	***p***	**OR**_**Meta **_**(95% CI)**^**#**^	**I**^**2**^
rs10202709	GG	864	651	1		1	
	GA	61	40	1.13 (0.74-1.72)	0.583	1.15 (0.63-2.10)	47.99
	AA	3	2	1.15 (0.19-711)	0.881	1.13 (0.18-7.09)	0
	G	0.96	0.97	1		1	
	A	0.04	0.03	1.24 (0.76-1.67)	0.565	1.16 (0.72-1.87)	23.50
rs6725958	CC	233	213	1		1	
	CA	489	355	1.17 (0.92-1.48)	0.201	1.08 (0.76-1.53)	0
	AA	206	125	1.39 (1.03-1.88)	0.030	1.40 (1.04-1.89)	0
	C	0.52	0.56	1		1	
	A	0.48	0.44	1.17 (1.01-1.35)	0.033	1.17 (1.01-1.35)	0
rs940739	AA	550	468	1		1	
	AT	337	198	1.44 (1.16-1.80)	0.001&	1.46 (1.17-1.83)	0
	TT	41	27	1.14 (0.68-1.91)	0.624	1.11 (0.67-1.86)	0
	A	0.77	0.82	1		1	
	T	0.23	0.18	1.28 (1.07-1.53)	0.008	1.28 (1.07-1.54)	0
rs2304573	GG	529	385	1		1	
	GA	335	256	0.97 (0.78-1.20)	0.754	0.97 (0.78-1.21)	0
	AA	64	52	0.93 (0.62-1.38)	0.701	0.94 (0.63-1.40)	0
	G	0.75	0.74	1		1	
	A	0.25	0.26	0.96 (0.82-1.13)	0.647	0.97 (0.82-1.14)	0
rs11651	AA	441	305	1		1	
	AG	383	294	0.90 (0.73-1.12)	0.359	0.91 (0.73-1.13)	0
	GG	104	94	0.78 (0.57-1.08)	0.138	0.78 (0.57-1.08)	0
	A	0.68	0.65	1		1	
	G	0.32	0.35	0.88 (0.76-1.03)	0.112	0.89 (0.76-1.04)	0
rs3796123	TT	768	577	1		1	
	TA	148	112	0.95 (0.72-1.25)	0.691	0.96 (0.73-1.27)	0
	AA	12	4	2.14 (0.67-6.87)	0.202	2.14 (0.38-12.14)	44.49
	T	0.91	0.91	1		1	
	A	0.09	0.09	1.03 (0.80-1.33)	0.818	1.04 (0.75-1.44)	36.03

^#^Data have been adjusted by gender, age and BMI; &*p*-values were based upon Bonferroni’s method.

### Association analyses for ITGAV polymorphisms in the combined studies

Genotypic and allelic distributions of the four *ITGAV* SNPs and their associations with knee OA risk are shown in Table 4. No deviation from the HWE was observed in the controls (*p* > 0.05). There was no significant genotypic or allelic association identified between the knee OA cases and healthy controls with the four *ITGAV* SNPs in both study cohorts. However, when data from the two independent studies were combined, rs10174098A/G was associated with a lower risk of knee OA (adjusted OR = 0.77, 95% CI = 0.62–0.96, *p* = 0.020).

**Table 4:** **Genotype distributions of *ITGAV *polymorphisms and their association with OA risk**

**SNP**		**Study 1**			**Study 2**			**Study 1 + Study 2**		
		**Case**	**Control**	**OR (95% CI)**^**#**^	**Case**	**Control**	**OR (95% CI)**^**#**^	**OR (95% CI)**^**#$**^	**OR**_**Meta**_	**I**^**2**^
rs3911238	GG	294	216	1	381	266	1	1	1	
	GC	99	89	0.84 (0.59-1.82)	129	101	0.86 (0.63-1.18)	0.84 (0.67-1.06)	0.85 (0.67-1.08)	0
	CC	10	9	0.92 (0.36-2.40)	15	12	0.92 (0.42-2.03)	0.93 (0.51-1.72)	0.92 (0.50-1.68)	0
	G	0.85	0.83	1	0.85	0.84	1	1	1	
	C	0.15	0.17	0.87 (0.65-1.17)	0.15	0.16	0.89 (0.69-1.16)	0.88 (0.73-1.07)	0.88 (0.73-1.07)	0
rs10174098	AA	285	209	1	351	240	1	1	1	
	AG	104	96	0.74 (0.52-1.04)	154	129	0.77 (0.57-1.03)	0.77 (0.62-0.96)	0.76 (0.60-0.95)	0
	GG	14	9	1.27 (0.53-3.05)	20	10	1.19 (0.53-2.64)	1.22 (0.68-2.20)	1.23 (0.68-2.22)	0
	A	0.84	0.82	1	0.82	0.80	1	1	1	
	G	0.16	0.18	0.86 (0.65-1.14)	0.18	0.20	0.87 (0.68-1.11)	0.88 (0.73-1.05)	0.87 (0.72-1.04)	0
rs3738919	CC	366	273	1	468	344	1	1	1	
	CA	35	37	0.66 (0.39-1.09)	54	44	0.87 (0.56-1.34)	0.77 (0.55-1.06)	0.78 (0.55-1.09)	0
	AA	2	4	0.29 (0.05-1.70)	3	1	2.18 (0.22-21.58)	0.70 (0.19-2.50)	0.69 (0.10-4.90)	46.58
	C	0.95	0.93	1	0.94	0.94	1	1	1	
	A	0.05	0.07	0.60 (0.38-0.95)	0.06	0.06	0.93 (0.62-1.40)	0.77 (0.57-1.04)	0.76 (0.49-1.16)	49.44
rs1448427	AA	267	196	1	325	234	1	1	1	
	AG	123	108	0.90 (0.37-2.18)	179	129	1.00 (0.49-2.03)	0.91 (0.74-1.13)	0.96 (0.55-1.67)	0
	GG	13	10	0.79 (0.57-1.10)	21	16	1.00 (0.75-1.34)	0.96 (0.55-1.66)	0.90 (0.72-1.13)	11.32
	A	0.82	0.80	1	0.79	0.79	1	1	1	
	G	0.18	0.20	0.85 (0.65-1.12)	0.21	0.21	1.00 (0.79-1.27)	0.94 (0.79-1.12)	0.93 (0.78-1.11)	0

^#^Data have been adjusted by gender, age and BMI; ^$^Simply pooled OR.

In males, significant associations were observed between *ITGAV* rs3738919C/A and OA (Additional file 1: Table S4), but no associations were found between OA and *FN-1* polymorphisms among males (Additional file 1: Table S2). In females, genotypic and allelic frequencies of *FN-1* rs6725958C/A and rs940739A/T and *ITGAV* rs10174098A/G polymorphisms differed significantly between knee OA cases and healthy controls (Additional file 1: Tables S3 and S4). Additionally, the dominant mode of *FN-1* rs640739 also appeared to be associated with a significantly higher risk of knee OA (adjusted OR = 1.41, 95% CI = 1.14–1.74, *p* = 0.002) (Additional file 1: Table S5).

In the recessive mode, the 10 *FN-1* and *ITGAV* SNPs showed no significance (Additional file 1: Table S6). After correcting for multiple comparisons, no SNPs appeared to be associated with OA. We further analyzed interactions between *FN-1* rs940739A/T and obesity, but observed no significant interaction between the two (*p* for interaction = 0.15 for the interaction term, Additional file 1: Table S7).

### Stratification analysis according to disease severity

Stratifying the patients by KL score identified significant differences in *FN-1* rs6725958C/A alleles and genotypes between patients with both grade 3 and 4 knee OA and controls (AA/CC, adjusted OR = 2.11, 95% CI = 1.45–3.06; adjusted OR = 0.54, 95% CI = 0.36–0.82; CA/CC, adjusted OR = 1.58, 95% CI = 1.16–2.16). *FN-1* rs940739A/T was also associated with grade 4 knee OA (AT/AA, adjusted OR = 2.07, 95% CI = 1.59–2.69) (Table 5).

**Table 5:** **Association of *FN-1 *rs6725958 and rs940739 polymorphisms among different grade OA cases and controls**

**Genetic risk factor**	**Genotype**	**Cases**	**Model**	**K/L score**^**$**^		
				**K/L**	**Adjusted OR (95% CI)**^**#**^	***p***
rs6725958						
	CC	233	AA/CC	2	1.10 (0.74-1.65)	0.628
	CA	489	AA/CC	3	0.81 (0.49-1.35)	0.418
	AA	206	AA/CC	4	2.11 (1.45-3.06)	< 0.001
			CA/CC	2	1.14 (0.83-1.55)	0.427
			CA/CC	3	0.54 (0.36-0.82)	0.003
			CA/CC	4	1.58 (1.16-2.16)	0.004
rs940739						
	AA	550	TT/AA	2	1.12 (0.59-2.14)	0.729
	AT	337	TT/AA	3	1.37 (0.59-3.18)	0.465
	TT	41	TT/AA	4	1.15 (0.59-2.24)	0.668
			TA/AA	2	0.96 (0.71-1.30)	0.801
			TA/AA	3	1.22 (0.82-1.18)	0.339
			TA/AA	4	2.07 (1.59-2.69)	< 0.001

^$^Grade 0, 1 as a reference category; OA cases = study 1 + study 2; ^#^Data have been adjusted by gender, age and BMI.

### Haplotype analysis of FN-1

Linkage disequilibrium analysis confirmed disequilibrium between SNPs rs2304573 and rs11651 (r^2^ = 0.73; data not shown). The haplotype analysis of *FN-1* polymorphisms in knee OA cases and control subjects is shown in Table 6. The frequency of haplotype AAG was 6.6% in knee OA patients compared with 10.1% in controls (OR = 0.63, 95% CI = 0.49–0.81, *p* < 0.001). By contrast, haplotypes TGA and TAA were more common in knee OA patients (4.7% and 2.2%, respectively) than controls (2.4% and 1.0%, respectively) (OR = 2.03 and 2.12, 95% CI = 1.35–3.06 and 1.16–3.89, *p* < 0.001 and = 0.013, respectively).

**Table 6:** **Haplotype analysis of the three identified SNPs in *FN-1 *between OA cases and control subjects in both studies**

**Haplotype**			**Frequencies**		***p***	**OR (95% CI)**
**rs940739**	**rs2304573**	**rs11651**	**Case**	**Control**		
A	G	A	0.581	0.597	0.386	0.94 (0.82-1.08)
T	G	G	0.130	0.127	0.771	1.03 (0.84-1.27)
A	G	G	0.095	0.099	0.729	0.96 (0.76-1.21)
A	A	G	0.066	0.101	< 0.001	0.63 (0.49-0.81)
T	G	A	0.047	0.024	< 0.001	2.03 (1.35-3.06)
A	A	A	0.031	0.022	0.087	1.47 (0.94-2.30)
T	G	G	0.027	0.021	0.289	1.28 (0.81-2.03)
T	A	A	0.022	0.010	0.013	2.12 (1.16-3.89)

## Discussion

After conducting two independent studies involving the genotyping and evaluation of disease severity of 928 knee OA patients and 693 healthy controls from a Han Chinese population, we have identified an association between the *FN-1* rs940739A/T SNP and OA. When data from both studies were combined, we also identified an association between the *ITGAV* rs10174098A/G SNP and knee OA.

Several recent studies have identified *FN-1* mutations and polymorphisms that affect host susceptibility to a variety of diseases including calcium oxalate stone disease [21], schizophrenia [22], lung cancer [23], and fibrosing alveolitis in systemic sclerosis [24]*.* However, no previous studies have reported on an association between *FN-1* polymorphisms and knee OA. The current study has, for the first time, revealed that the *FN-1* intronic polymorphism rs940739A/T is associated with knee OA. SNPs in introns are known to influence the regulation of transcription, splicing, and other aspects of RNA processing or stability [25,26]. For instance, the intronic SNPs in *FTO *[27], *COL1A1 *[28], and *BANK1 *[29] affect primary transcript levels while the risk allele of the intronic SNP in the prothrombin gene may influence splicing efficiency [30]. However, it is currently unclear whether the intronic SNP identified as being associated with knee OA in the current study performs a functional role by exerting a direct effect on *FN-1* expression or whether it is in linkage disequilibrium with another functional SNP.

The role of FN and FN-fs in the development of knee OA is well recognized and supported by a number of studies. FN-fs, the central cell-, NH2-terminal heparin-, and NH2-terminal gelatin-binding fragments of FN have been shown to stimulate cartilage chondrolysis by inhibiting anabolic activity while increasing production of matrix metalloproteinases [31]. Importantly, *FN-1* expression appears to be differentially regulated in monolayer cultured chondrocytes from OA and normal donors [32].

When we analyzed the association of SNP expression with the radiological grade of knee OA, a significant relationship was seen in patients with KL grade 4 knee OA with genotypes rs6725958C/A and rs940739A/T, indicating that these *FN-1* polymorphisms are associated with advanced knee OA in the current Han Chinese population. The reasons for these associations are not clear. Genetic risk factors can influence the risk of development and progression of knee OA at various stages during the course of the disease. Because *FN-1* polymorphisms could contribute to the risk of OA at different phases or throughout the disease process, further functional studies on such polymorphisms are required.

Haplotype analysis demonstrated an association between *FN-1* and knee OA. Our data revealed that patients carrying TGA or TAA haplotypes had a higher risk of developing knee OA than those without, suggesting that multiple variants in *FN-1* affect the risk. The *FN-1* haplotype has previously been shown to have a significant influence on whether a fetus is at risk of being small-for-gestational-age [33], indicating that haplotype status could be important in tissue homeostasis.

Fibronectin and Fn-fs interact with chondrocytes and other joint tissue cells through integrins by which they can regulate cell function through a variety of intracellular signal cascades [5,34,35]. Although the α5β1 integrin is the major fibronectin receptor expressed by chondrocytes [7], αV is also abundantly expressed by articular chondrocytes; however, its biological significance in cartilage has been less well studied. *ITGAV* polymorphisms are associated with a number of conditions including rheumatoid arthritis [36], chronic hepatitis B virus infection [37], and primary biliary cirrhosis [38]. In the current study, the *ITGAV* SNP rs10174098A/G was only associated with knee OA when both study data were combined. Thus it is not entirely clear whether this weaker association with OA is genuine. Further independent studies need to be undertaken to clarify the situation.

## Conclusion

The present study suggests that there is a significant association with the *FN-1* polymorphism rs940739A/T and susceptibility to knee OA in a Chinese Han population. Such polymorphisms may explain why some individuals are at a higher risk for knee OA. Further studies on other larger populations combined with *in vitro* functional analyses are now crucial to elucidate the effects of the polymorphism on knee OA pathogenesis and susceptibility.

## Competing interests

The authors declare that they have no competing interests.

## Authors’ contributions

HYY conceived, designed, and performed the experiments, analyzed the data, and wrote the paper. SLS conceived and designed the experiments, analyzed the data, and contributed reagents/materials/analysis tools. HSL conceived, designed and performed the experiments, contributed reagents/materials/analysis tools, and wrote the paper. CHL conceived, designed and performed the experiments, contributed reagents/materials/analysis tools, and wrote the paper. YJP performed the experiments, and analyzed the data. CCW performed the experiments, and analyzed the data. DMS wrote the paper. All authors critically revised the manuscript and approved the final version.

## Pre-publication history

The pre-publication history for this paper can be accessed here:

http://www.biomedcentral.com/1471-2474/15/173/prepub

## Supplementary Material

Additional file 1: Table S1 Estimation of statistical power for the present study. **Table S2.** Genotype distributions and allele frequencies for the FN gene polymorphisms in male OA patients and healthy control groups. **Table S3.** Genotype distributions and allele frequencies for the FN gene polymorphisms in female OA patients and healthy control groups. **Table S4.** Genotype distributions and allele frequencies of the ITGAV gene by gender. **Table S5.** Analyses of the association of 10 SNPs in FN and ITGAV gene with OA (dominant model). **Table S6.** Analyses of the association of 10 SNPs in FN and ITGAV gene with OA (recessive model). **Table S7.** Joint effects of FN rs940739 and obesity among 928 cases of OA and 693 control subjects. Click here for file
