# Supplementary material for: An intron polymorphism of the fibronectin gene is associated with end-stage knee osteoarthritis in a Han Chinese population: two independent case-control studies
Source: BMC Musculoskelet Disord. 2014 May 23;15:173. doi: 10.1186/1471-2474-15-173 (PMC4050217; doi:10.1186/1471-2474-15-173)
Supplement: Additional file 1: Table S1 — Estimation of statistical power for the present study. Table S2. Genotype distributions and allele frequencies for the FN gene polymorphisms in male OA patients and healthy control groups. Table S3. Genotype distributions and allele frequencies for the FN gene polymorphisms in female OA patients and healthy control groups. Table S4. Genotype distributions and allele frequencies of the ITGAV gene by gender. Table S5. Analyses of the association of 10 SNPs in FN and ITGAV gene with OA (dominant model). Table S6. Analyses of the association of 10 SNPs in FN and ITGAV gene with OA (recessive model). Table S7. Joint effects of FN rs940739 and obesity among 928 cases of OA and 693 control subjects. [file 1471-2474-15-173-S1.doc]

Table S1 Estimation of statistical power for the present study

|  | SNP | rs10202709 | rs6725958 | rs940739 | rs2304573 | rs11651 | rs3796123 | rs3911238 | rs10174098 | rs3738919 | rs1448427 |
| --- | --- | --- | --- | --- | --- | --- | --- | --- | --- | --- | --- |
| OR | EAF | (0.04) | (0.48) | (0.23) | (0.25) | (0.32) | (0.09) | (0.15) | (0.16) | (0.05) | (0.18) |
| 1.10 |  | 0.10 | 0.32 | 0.22 | 0.27 | 0.30 | 0.15 | 0.20 | 0.21 | 0.11 | 0.23 |
| 1.30 |  | 0.37 | 0.96 | 0.93 | 0.94 | 0.96 | 0.72 | 0.87 | 0.88 | 0.51 | 0.90 |
| 1.50 |  | 0.71 | 1.00 | 1.00 | 1.00 | 1.00 | 0.95 | 1.00 | 1.00 | 0.88 | 1.00 |
| 1.70 |  | 0.97 | 1.00 | 1.00 | 1.00 | 1.00 | 1.00 | 1.00 | 1.00 | 0.98 | 1.00 |
| 2.00 |  | 1.00 | 1.00 | 1.00 | 1.00 | 1.00 | 1.00 | 1.00 | 1.00 | 1.00 | 1.00 |
| 2.50 |  | 1.00 | 1.00 | 1.00 | 1.00 | 1.00 | 1.00 | 1.00 | 1.00 | 1.00 | 1.00 |

The prevalence of OA assumed to be 15%, α = 0.05

Table S2 Genotype distributions and allele frequencies for the FN gene polymorphisms in male OA patients and healthy control groups

| SNP |  | Case | Control | Crude OR  (95% CI) | *p* | Adjusted OR (95% CI) ^+^ | *p* |
| --- | --- | --- | --- | --- | --- | --- | --- |
| rs10202709 | GG | 299 | 286 | 1 | 0.39 | 1 |  |
|  | GA | 17 | 16 | 1.02 (0.50-2.05) |  | 1.00 (0.49-2.02) | 0.99 |
|  | AA | 2 | 0 | - |  | - |  |
|  | G | 0.97 | 0.97 | 1 | 0.53 | 1 |  |
|  | A | 0.03 | 0.03 | 1.26 (0.56-2.43) |  | 1.23 (0.63-2.38) | 0.55 |
| rs6725958 | CC | 89 | 84 | 1 | 0.95 | 1 |  |
|  | CA | 168 | 157 | 1.01 (0.70-1.46) |  | 1.01 (0.70-1.46) | 0.97 |
|  | AA | 61 | 61 | 0.94 (0.59-1.50) |  | 0.94 (0.59-1.50) | 0.80 |
|  | C | 0.54 | 0.54 | 1 | 0.83 | 1 |  |
|  | A | 0.46 | 0.46 | 0.98 (0.78-1.22) |  | 0.98 (0.78-1.22) | 0.83 |
| rs940739 | AA | 197 | 206 | 1 | 0.24 | 1 |  |
|  | AT | 106 | 82 | 1.35 (0.95-1.92) |  | 1.39 (0.98-1.97) | 0.07 |
|  | TT | 15 | 14 | 1.12 (0.53-2.38) |  | 1.07 (0.50-2.28) | 0.86 |
|  | A | 0.79 | 0.82 | 1 | 0.16 | 1 |  |
|  | T | 0.21 | 0.18 | 1.22 (0.92-1.62) |  | 1.23 (0.92-1.62) | 0.16 |
| rs2304573 | GG | 191 | 162 | 1 | 0.27 | 1 |  |
|  | GA | 106 | 117 | 0.77 (0.55-1.08) |  | 0.78 (0.56-1.09) | 0.15 |
|  | AA | 21 | 23 | 0.77 (0.41-1.45) |  | 0.79 (0.42-1.48) | 0.45 |
|  | G | 0.77 | 0.73 | 1 | 0.13 | 1 |  |
|  | A | 0.23 | 0.27 | 0.82 (0.63-1.06) |  | 0.83 (0.64-1.07) | 0.16 |
| rs11651 | AA | 150 | 136 | 1 | 0.85 | 1 |  |
|  | AG | 128 | 125 | 0.93 (0.66-1.30) |  | 0.94 (0.67-1.32) | 0.70 |
|  | GG | 40 | 41 | 0.89 (0.54-1.45) |  | 0.90 (0.55-1.47) | 0.69 |
|  | A | 0.67 | 0.66 | 1 | 0.56 | 1 |  |
|  | G | 0.33 | 0.34 | 0.93 (0.74-1.18) |  | 0.94 (0.74-1.19) | 0.61 |
| rs3796123 | TT | 281 | 255 | 1 | 0.34 | 1 |  |
|  | TA | 36 | 45 | 0.73 (0.45-1.16) |  | 0.72 (0.45-1.16) | 0.18 |
|  | AA | 1 | 2 | 0.45 (0.04-5.03) |  | 0.44 (0.04-4.86) | 0.50 |
|  | T | 0.94 | 0.92 | 1 | 0.14 | 1 |  |
|  | A | 0.06 | 0.08 | 0.72 (0.46-1.12) |  | 0.72 (0.46-1.11) | 0.14 |

^+^Data have been adjusted by age and BMI.

Table S3 Genotype distributions and allele frequencies for the FN gene polymorphisms in female OA patients and healthy control groups

| SNP |  | Case | Control | Crude OR  (95% CI) | *p* | Adjusted OR (95% CI) ^+^ | *p* |
| --- | --- | --- | --- | --- | --- | --- | --- |
| rs10202709 | GG | 561 | 365 | 1 | 0.53 | 1 |  |
|  | GA | 43 | 24 | 1.17 (0.70-1.95) |  | 1.29 (0.74-2.24) | 0.37 |
|  | AA | 1 | 2 | 0.33 (0.03-3.60) |  | 0.37 (0.03-4.47) | 0.43 |
|  | G | 0.96 | 0.96 | 1 | 0.87 | 1 |  |
|  | A | 0.04 | 0.04 | 1.04 (0.64-1.68) |  | 1.14 (0.69-1.91) | 0.61 |
| rs6725958 | CC | 143 | 129 | 1 | 0.001 | 1 |  |
|  | CA | 319 | 198 | 1.45 (1.08-1.96) |  | 1.23 (0.90-1.69) | 0.19 |
|  | AA | 143 | 64 | 2.02 (1.38-2.94) |  | 1.71 (1.15-2.55) | 0.008 |
|  | C | 0.50 | 0.58 | 1 | < 0.001 | 1 |  |
|  | A | 0.50 | 0.42 | 1.39 (1.17-1.68) |  | 1.28 (1.06-1.55) | 0.01 |
| rs940739 | AA | 351 | 262 | 1 | 0.02 | 1 |  |
|  | AT | 229 | 116 | 1.47 (1.12-1.94) |  | 1.44 (1.08-1.93) | 0.01 |
|  | TT | 25 | 13 | 1.44 (0.72-2.86) |  | 1.23 (0.60-2.54) | 0.58 |
|  | A | 0.77 | 0.82 | 1 | 0.009 | 1 |  |
|  | T | 0.23 | 0.18 | 1.35 (1.08-1.69) |  | 1.30 (1.02-1.64) | 0.03 |
| rs2304573 | GG | 336 | 223 | 1 | 0.81 | 1 |  |
|  | GA | 227 | 139 | 1.08 (0.83-1.42) |  | 1.11 (0.84-1.48) | 0.46 |
|  | AA | 42 | 29 | 0.96 (0.58-1.59) |  | 1.01 (0.59-1.71) | 0.98 |
|  | G | 0.74 | 0.75 | 1 | 0.80 | 1 |  |
|  | A | 0.26 | 0.25 | 1.03 (0.84-1.26) |  | 1.05 (0.85-1.31) | 0.64 |
| rs11651 | AA | 289 | 169 | 1 | 0.22 | 1 |  |
|  | AG | 252 | 169 | 0.87 (0.66-1.15) |  | 0.86 (0.65-1.15) | 0.30 |
|  | GG | 64 | 53 | 0.71 (0.47-1.07) |  | 0.67 (0.44-1.04) | 0.07 |
|  | A | 0.69 | 0.65 | 1 | 0.08 | 1 |  |
|  | G | 0.31 | 0.35 | 0.84 (0.70-1.02) |  | 0.83 (0.68-1.01) | 0.06 |
| rs3796123 | TT | 484 | 322 | 1 | 0.81 | 1 |  |
|  | TA | 110 | 67 | 1.09 (0.78-1.53) |  | 1.08 (0.76-1.54) | 0.66 |
|  | AA | 11 | 2 | 3.66 (0.81-16.62) |  | 4.11 (0.82-20.76) | 0.09 |
|  | T | 0.91 | 0.09 | 1 | 0.19 | 1 |  |
|  | A | 0.89 | 0.11 | 1.23 (0.91-1.66) |  | 1.22 (0.89-1.69) | 0.22 |

^+^Data have been adjusted by age and BMI.

**Table S4 Genotype distributions and allele frequencies of the ITGAV gene by gender**

|  | SNP |  | Case | Control | Adjusted OR (95% CI) ^+^ | MAF^#^ (case/control) |
| --- | --- | --- | --- | --- | --- | --- |
| Female |  |  |  |  |  |  |
|  | rs3911238 | GG | 435 | 265 | 1 | 0.16/0.18 |
|  |  | GC | 152 | 112 | 0.82 (0.60-1.10) |  |
|  |  | CC | 18 | 14 | 1.03 (0.48-2.23) |  |
|  | rs10174098 | AA | 417 | 251 | 1 | 0.17/0.19 |
|  |  | AG | 165 | 127 | 0.72 (0.54-0.97) |  |
|  |  | GG | 23 | 13 | 1.11 (0.53-2.31) |  |
|  | rs3738919 | CC | 537 | 347 | 1 | 0.06/0.06 |
|  |  | CA | 65 | 41 | 1.01 (0.65-1.57) |  |
|  |  | AA | 3 | 3 | 0.46 (0.09-2.35) |  |
|  | rs1448427 | AA | 375 | 226 | 1 | 0.21/0.24 |
|  |  | AG | 206 | 146 | 0.85 (0.64-1.13) |  |
|  |  | GG | 24 | 19 | 0.84 (0.43-1.65) |  |
| Male |  |  |  |  |  |  |
|  | rs3911238 | GG | 237 | 217 | 1 | 0.14/0.15 |
|  |  | GC | 74 | 78 | 0.87 (0.60-1.26) |  |
|  |  | CC | 7 | 7 | 0.90 (0.31-2.62) |  |
|  | rs10174098 | AA | 217 | 198 | 1 | 0.17/0.18 |
|  |  | AG | 91 | 98 | 0.83 (0.59-1.17) |  |
|  |  | GG | 10 | 6 | 1.49 (0.53-4.17) |  |
|  | rs3738919 | CC | 292 | 260 | 1 | 0.04/0.07 |
|  |  | CA | 24 | 40 | 0.52 (0.31-0.89) |  |
|  |  | AA | 2 | 2 | 0.98 (0.14-7.00) |  |
|  | rs1448427 | AA | 214 | 204 | 1 | 0.18/0.17 |
|  |  | AG | 95 | 91 | 1.00 (0.71-1.42) |  |
|  |  | GG | 9 | 7 | 1.26 (0.46-3.44) |  |

^+^Data have been adjusted by age and BMI. ^#^MAF (The minor allele frequency)

Table S5 Analyses of the association of 10 SNPs in FN and ITGAV gene with OA (dominant model)

| SNP |  | Case | Control | Crude OR  (95% CI) | Adjusted OR (95% CI) ^+^ | *p* |
| --- | --- | --- | --- | --- | --- | --- |
| rs10202709 | GG | 864 | 651 | 1 | 1 |  |
|  | GA+AA | 64 | 42 | 1.15 (0.77-1.72) | 1.13 (0.75-1.71) | 0.57 |
| rs6725958 | CC | 233 | 213 | 1 | 1 |  |
|  | CA+AA | 695 | 480 | 1.32 (1.06-1.65)^*^ | 1.23 (0.98-1.54) | 0.08 |
| rs940739 | AA | 550 | 468 | 1 | 1 |  |
|  | AT+TT | 378 | 225 | 1.43 (1.16-1.76) ^*^ | 1.41 (1.14-1.74) | 0.002 |
| rs2304573 | GG | 529 | 385 | 1 | 1 |  |
|  | GA+AA | 399 | 308 | 0.94 (0.77-1.15) | 0.96 (0.78-1.18) | 0.69 |
| rs11651 | AA | 441 | 305 | 1 | 1 |  |
|  | AG+GG | 487 | 388 | 0.87 (0.71-1.06) | 0.87 (0.71-1.07) | 0.20 |
| rs3796123 | TT | 768 | 577 | 1 | 1 |  |
|  | TA+ AA | 160 | 116 | 1.04 (0.80-1.35) | 0.92 (0.75-1.29) | 0.92 |
| rs3911238 | GG | 675 | 482 | 1 | 1 |  |
|  | GC+CC | 253 | 211 | 0.86 (0.69-1.06) | 0.85 (0.68-1.07) | 0.16 |
| rs10174098 | AA | 636 | 449 | 1 | 1 |  |
|  | AG+GG | 292 | 244 | 0.85 (0.69-1.04) | 0.80 (0.65-1.00) | 0.05 |
| rs3738919 | CC | 834 | 607 | 1 | 1 |  |
|  | CA+AA | 94 | 86 | 0.80 (0.58-1.09) | 0.76 (0.55-1.05) | 0.10 |
| rs1448427 | AA | 592 | 430 | 1 | 1 |  |
|  | AG+GG | 336 | 263 | 0.93 (0.76-1.14) | 0.92 (0.74-1.13) | 0.42 |

^+^Adjusted for age, sex, and the body mass index (BMI); **p* < 0.05

Table S6 Analyses of the association of 10 SNPs in FN and ITGAV gene with OA (recessive model)

| SNP |  | Case | Control | Crude OR  (95% CI) | Adjusted OR (95% CI) ^+^ | *p* |
| --- | --- | --- | --- | --- | --- | --- |
| rs10202709 | GG+GA | 925 | 691 | 1 | 1 |  |
|  | AA | 3 | 2 | 1.21 (0.19-6.72) | 1.14 (0.19-7.05) | 0.89 |
| rs6725958 | CC+CA | 722 | 568 | 1 | 1 |  |
|  | AA | 206 | 125 | 1.30 (1.01-1.66) | 1.26 (0.96-1.62) | 0.08 |
| rs940739 | AA+AT | 887 | 666 | 1 | 1 |  |
|  | TT | 41 | 27 | 1.14 (0.69-1.87) | 1.01 (0.60-1.68) | 0.97 |
| rs2304573 | GG+GA | 864 | 641 | 1 | 1 |  |
|  | AA | 64 | 52 | 0.91 (0.62-134) | 0.94 (0.63-1.39) | 0.75 |
| rs11651 | AA+AG | 824 | 599 | 1 | 1 |  |
|  | GG | 104 | 94 | 0.80 (0.60-1.01) | 0.83 (0.61-1.12) | 0.21 |
| rs3796123 | TT+TA | 916 | 689 | 1 | 1 |  |
|  | AA | 12 | 4 | 2.28 (0.73-703) | 2.16 (0.67-6.92) | 0.20 |
| rs3911238 | GG+GC | 903 | 672 | 1 | 1 |  |
|  | CC | 25 | 21 | 0.89 (0.49-1.60) | 0.98 (0.53-1.79) | 0.94 |
| rs10174098 | AA+AG | 894 | 674 | 1 | 1 |  |
|  | GG | 34 | 19 | 1.35 (0.76-2.39) | 1.32 (0.74-2.38) | 0.35 |
| rs3738919 | CC+CA | 923 | 688 | 1 | 1 |  |
|  | AA | 5 | 5 | 0.75 (0.22-2.59) | 0.72 (0.20-2.57) | 0.61 |
| rs1448427 | AA+AG | 894 | 677 | 1 | 1 |  |
|  | GG | 34 | 26 | 0.98 (0.58-1.64) | 0.99 (0.57-1.70) | 0.96 |

^+^Adjusted for age, sex, and the body mass index (BMI); **p* < 0.05

Table S7 Joint effects of FN rs940739 and obesity among 928 cases of OA and 693 control subjects.

| Risk factor | FN  rs940739 A/T | case | control | ^+^Adjusted OR (95%CI) | *p*-value |
| --- | --- | --- | --- | --- | --- |
| BMI ≦ 27 | AA | 447 | 393 | 1 (reference) |  |
|  | AT | 264 | 159 | 1.65 (1.33-2.04) | < 0.001 |
|  | TT | 37 | 21 | 1.15 (0.72-1.85) | 0.56 |
| BMI > 27 | AA | 103 | 74 | 0.39 (0.27-0.56) | < 0.001 |
|  | AT | 73 | 38 | 0.62 (0.39-0.97) | 0.04 |
|  | TT | 4 | 6 | 0.16 (0.06-0.47) | 0.001 |
| *p*-interaction = 0.15 | | |  |  |  |

^+^Adjusted for age, sex, and the BMI; **p* < 0.05
